# Value of [^68^Ga]Ga-somatostatin receptor PET/CT in the grading of pulmonary neuroendocrine (carcinoid) tumours and the detection of disseminated disease: single-centre pathology-based analysis and review of the literature

**DOI:** 10.1186/s13550-022-00900-3

**Published:** 2022-05-07

**Authors:** Anne-Leen Deleu, Annouschka Laenen, Herbert Decaluwé, Birgit Weynand, Christophe Dooms, Walter De Wever, Sander Jentjens, Karolien Goffin, Johan Vansteenkiste, Koen Van Laere, Paul De Leyn, Kristiaan Nackaerts, Christophe M. Deroose

**Affiliations:** 1 Nuclear Medicine, University Hospitals Leuven, Herestraat 49, 3000 Louvain, Belgium; 2 Interuniversity Institute for Biostatistics and Statistical Bioinformatics, Louvain, Belgium; 3 Thoracic Surgery, University Hospitals Leuven, Louvain, Belgium; 4 Pathology, University Hospitals Leuven, Louvain, Belgium; 5 Department of Respiratory Diseases and Respiratory Oncology Unit, University Hospitals Leuven, Louvain, Belgium; 6 Radiology, University Hospitals Leuven, Louvain, Belgium; 7 Nuclear Medicine and Molecular Imaging, Department of Imaging and Pathology KU Leuven, Louvain, Belgium

**Keywords:** Neuroendocrine tumour, Pulmonary carcinoid, Bronchial carcinoid, Typical carcinoid, Atypical carcinoid, PET, Somatostatin receptor, [^68^Ga]Ga-DOTATATE, [^68^Ga]Ga-DOTATOC, [^18^F]FDG

## Abstract

**Background:**

Although most guidelines suggest performing a positron emission tomography/computed tomography (PET/CT) with somatostatin receptor (SSTR) ligands for staging of pulmonary carcinoid tumours (PC), only a limited number of studies have evaluated the role of this imaging tool in this specific patient population. The preoperative differentiation between typical carcinoid (TC) and atypical carcinoid (AC) and the extent of dissemination (N/M status) are crucial factors for treatment allocation and prognosis of these patients. Therefore, we performed a pathology-based retrospective analysis of the value of SSTR PET/CT in tumour grading and detection of nodal and metastatic involvement of PC and compared this with the previous literature and with [^18^F]FDG PET/CT in a subgroup of patients.

**Methods:**

SSTR PET/CT scans performed between January 2007 and May 2020 in the context of PC were included. If available, [^18^F]FDG PET/CT images were also evaluated. The maximum standardized uptake (SUV_max_) values of the primary tumour, of the pathologically examined hilar and mediastinal lymph node stations, as well as of the distant metastases, were recorded. Tumoural SUV_max_ values were related to the tumour type (TC versus AC) for both SSTR and [^18^F]FDG PET/CT in diagnosing and differentiating both tumour types. Nodal SUV_max_ values were compared to the pathological status (N^+^ versus N^−^) to evaluate the diagnostic accuracy of SSTR PET/CT in detecting lymph node involvement. Finally, a mixed model analysis of all pathologically proven distant metastatic lesions was performed.

**Results:**

A total of 86 SSTR PET/CT scans performed in 86 patients with PC were retrospectively analysed. [^18^F]FDG PET/CT was available in 46 patients. Analysis of the SUV_max_ values in the primary tumour showed significantly higher SSTR uptake in TC compared with AC (median SUV_max_ 18.4 vs 3.8; *p* = 0.003) and significantly higher [^18^F]FDG uptake in AC compared to TC (median SUV_max_ 5.4 vs 3.5; *p* = 0.038). Receiver operating characteristic (ROC) curve analysis resulted in an area under the curve (AUC) of 0.78 for the detection of TC on SSTR PET/CT and of 0.73 for the detection of AC on [^18^F]FDG PET/CT. A total of 267 pathologically evaluated hilar and mediastinal lymph node stations were analysed. ROC analysis of paired SSTR/[^18^F]FDG SUV_max_ values for the detection of metastasis of TC in 83 lymph node stations revealed an AUC of 0.91 for SSTR PET/CT and of 0.74 for [^18^F]FDG PET/CT (difference 0.17; 95% confidence interval − 0.03 to 0.38; *p* = 0.10). In a sub-cohort of 10 patients with 12 distant lesions that were pathologically examined due to a suspicious aspect on SSTR PET/CT, a positive predictive value (PPV) of 100% was observed.

**Conclusion:**

Our findings confirm the higher SSTR ligand uptake in TC compared to AC and vice versa for [^18^F]FDG uptake. More importantly, we found a good diagnostic performance of SSTR PET/CT for the detection of hilar and mediastinal lymph node metastases of TC. Finally, a PPV of 100% for SSTR PET/CT was found in a small sub-cohort of patients with pathologically investigated distant metastatic lesions. Taken together, SSTR PET/CT has a very high diagnostic value in the TNM assessment of pulmonary carcinoids, particularly in TC, which underscores its position in European guidelines.

**Supplementary Information:**

The online version contains supplementary material available at 10.1186/s13550-022-00900-3.

## Background

Pulmonary carcinoid tumours (PC), also known as bronchial carcinoid tumours, are a group of well-differentiated, low (typical carcinoid, TC) to intermediate (atypical carcinoid, AC) grade neuroendocrine tumours (NETs), originating from enterochromaffin or Kulchitzky cells in the respiratory tract [1, 2]. Of all well-differentiated NETs, around 25% are located in the respiratory tract [1, 3, 4]. The age-adjusted incidence rate ranges from 0.2 to 2/100000 persons/year in both the USA and Europe [1–4]. There has been an increase in prevalence over the past decades, regardless of confounding demographic factors [1–3, 5]. Other types of pulmonary NETs include the high-grade large-cell neuroendocrine carcinoma (LCNEC) and small-cell lung carcinoma (SCLC), which are classified as different clinico-pathological entities, are characterized by the presence of tumour necrosis and higher mitotic rates and are associated with a worse prognosis [4, 6].

Pathologic examination is the cornerstone in the diagnostic assessment of any pulmonary NET, with the recent fifth edition of the WHO nomenclature of thoracic tumours being the current standard for classification [6]. Mitosis and necrosis are the histopathologic features that distinguish TC from AC [6, 7]. To provide the TNM staging for PC according to the eighth edition lung cancer stage classification, diagnostic tools vary widely between institutions and countries, with the traditional morphologic computed tomography (CT) still being the gold standard [2, 8]. However, CT imaging features of a PC are often nonspecific and are unable to differentiate between a PC, an adenocarcinoma or a squamous cell carcinoma, let alone between TC and AC [7, 9]. Also, a correct preoperative pathologic evaluation is generally hard to obtain, and the preoperative differentiation between TC and AC is considered not feasible even if using the Ki67 proliferation index [7, 10]. In this context, PET could be seen as a “noninvasive biopsy” and an accurate full TNM staging examination [7, 11].

Since several decades, it is known that NETs—in particular if well-differentiated—express high levels of somatostatin receptors (SSTR), specifically subtype 2 [12, 13]. In the early 1990s, the first studies with radiolabelled octreotide derivatives showed the potential for in vivo detection of SSTR expression within tumours, raising interest in this new molecular imaging tool [14]. [^111^In]In-pentetreotide (brand name: Octreoscan®) was one of the first widespread functional imaging tools for NETs [15, 16]. Over the past two decades, several other somatostatin analogs (SSA) with a higher affinity for SSTR have been developed (DOTATATE, DOTANOC, DOTATOC) [17]. These agents can be labelled with gallium-68 (^68^Ga), a positron emitter that enables PET imaging and thus provides improved image quality and spatial resolution [18, 19]. Moreover, novel agents that can be labelled with fluorine-18 or copper-64 will increase the availability of SSTR PET in the near future [20, 21]. SSTR PET/CT imaging proved to visualize nearly 80% of primary PC tumours [1]. Furthermore, it is the most sensitive imaging technique for the detection of metastatic disease, especially regarding bone metastases for which CT can have a low sensitivity [22–25]. Therefore, SSTR PET/CT is suggested as a basic tool in the TNM staging of PC in most guidelines, together with contrast-enhanced CT of the chest and liver in a late arterial phase [1, 26]. For [^18^F]FDG PET/CT, the detection rate of the primary tumour and metastatic lymph nodes from PC that are reported in the literature vary widely, probably due to the major difference in glucose metabolic activity between TC, AC and high-grade neuroendocrine lung tumours [7, 27]. In this way, the combined use of SSTR PET/CT and [^18^F]FDG PET/CT can be helpful in determining the biology of PC [1, 18, 28, 29].

Therapeutic strategies for PC include resection of the tumour for limited disease, administration of somatostatin analogs for carcinoid syndrome or as first-line systemic antiproliferative treatment in unresectable PCs, and systemic therapy for metastatic disease (everolimus, chemotherapy, peptide receptor radionuclide therapy (PRRT)) [1, 2, 4, 30].

In this study, we firstly wanted to study retrospectively the value of dual (SSTR/[^18^F]FDG) PET/CT in the assessment of the tumour biology, based on the widely known “flip-flop phenomenon” of high SSTR and low [^18^F]FDG uptake in well-differentiated tumours and the opposite imaging phenotype in poorly differentiated tumours [31]. Although hilar and mediastinal lymph node involvement is known as one of the most important prognostic factors in PC, no studies have been done to quantitatively assess the diagnostic performance of SSTR PET/CT in the detection of these regional lymph node metastases [32, 33]. The most common sites of distant PC metastases are the liver and bones. Therefore, we also wanted to evaluate the pathology-based diagnostic accuracy of SSTR PET/CT with specific emphasis on regional lymph node involvement and on distant metastatic disease, based on a retrospective analysis of the preoperative SSTR PET/CT images and the pathology reports. As most literature reviews on the diagnostic value of SSTR PET/CT focus on NETs in general, with a strong emphasis on gastroenteropancreatic (GEP) NETs [18], we also provide a literature overview of the diagnostic performance and impact of SSTR PET/CT on the TNM staging for pulmonary neuroendocrine tumours in particular.

## Methods

### Patient selection

This was a retrospective study with prior approval of the Research Ethics Committee UZ/KU Leuven (study number MP015178). Databases of the nuclear medicine department and the department of thoracic surgery were searched for patients who had a SSTR PET/CT and a standard CT in the context of staging or follow-up for a histologically proven NET of the lung in the period from January 2007 (start of SSTR PET/CT at our centre) to May 2020 (cut-off date). Only SSTR PET/CTs performed in our institution were included. The absence of an [^18^F]FDG PET/CT or the execution of [^18^F]FDG PET/CT in an external centre was not considered an exclusion criterion. Patient characteristics, clinical presentation, laboratory results, histopathology, diagnostic imaging, decision of the multidisciplinary tumour board, treatment and follow-up were noted.

### Positron emission tomography/computed tomography (PET/CT) protocols and image analysis

All SSTR PET/CTs were performed on a dedicated hybrid scanner (Biograph 16-slice HiRez LSO PET/CT (Siemens, Erlangen, Germany), Biograph 40 TruePoint PET/CT (Siemens, Erlangen, Germany) or Discovery MI4 PET/CT (GE, Milwaukee, WI, USA)). These cameras were EARL accredited for [^18^F]FDG and cross-calibrated for gallium-68. However, they are not accredited by the recently launched EARL accreditation programme for gallium-68. The tracer used was initially [^68^Ga]Ga-DOTATOC (synthesis as described) [34], with a switch to [^68^Ga]Ga-DOTATATE from the end of 2012 onwards. If an [^18^F]FDG PET/CT was performed during diagnostic work-up with a maximum interval of 6 months from the SSTR PET/CT, these data were retrieved for paired analysis. A paired analysis was only done if no therapeutic interventions occurred during the time interval between [^18^F]FDG and SSTR PET/CT. All images were re-evaluated using MIM software v 7.0 (MIM Software Inc., Cleveland, Ohio, USA) with annotation of the maximum standardized uptake value (SUV_max_) in the primary tumour, in all hilar and mediastinal lymph node stations that were pathologically evaluated and in each metastatic organ (distant lymph nodes, liver, bone, etc.). In patients with multiple metastatic lesions in one organ, the SUV_max_ of the most [^68^Ga]Ga-DOTA-SSA- or [^18^F]FDG-avid lesion was noted.

### Pathological evaluation

Systematic nodal dissection with histopathologic evaluation is part of the standard protocol during resections for PC in our centre, in line with the European Society of Thoracic Surgeons (ESTS) guidelines [35]. In agreement with the WHO criteria, the pathological specimens had been evaluated and classified as TC, AC or LCNEC based on the mitotic rate and the presence or absence of necrosis [6]. If resection or biopsy occurred in an external institution, we requested the reports and the classification as TC, AC or LCNEC was recorded. As only a single patient with LCNEC had the primary tumour in situ at the time of imaging, the tumoural SUV_max_ analysis was restricted to pathology-proven TC/AC tumours. Resected lymph nodes were labelled according to the “International Association for the Study of Lung Cancer (IASLC)” lymph node map [36]. The pathological evaluation of lymph nodes in each resected and examined mediastinal and/or hilar station, was noted for each resected station from the reports (N + /N-). For these lymph node stations, SUV_max_ values were calculated on the preoperative SSTR PET/CT, using the CT boundaries of the IASLC map.

### Statistical analysis

Frequencies and percentages were used to analyse qualitative variables, whereas medians were reported to descriptively analyse the quantitative variables. The Mann–Whitney test was used to test differences in tumoural SUV_max_ values between TCs and ACs for both SSTR and [^18^F]FDG PET/CT. A scatter plot was made to illustrate the association between paired tumoural SUV_max_ values (TC/AC) on [^18^F]FDG and SSTR PET/CT. The association of a metric and a dichotomous variable was analysed using receiver operating characteristics (ROC) curves, both for the detection of lymph node metastases as for the detection of TC on SSTR PET/CT and of AC on [^18^F]FDG PET/CT. The optimal cut-off SUV_max_ value to differentiate between AC and TC was defined by the maximal Youden’s index (sensitivity + specificity − 1) on the ROC curves of non-paired SUV_max_ values for the detection of TC on SSTR PET/CT and of AC on [^18^F]FDG PET/CT. To calculate sensitivity and specificity for *N* + disease from TC on SSTR PET/CT, an SUV_max_ cut-off value was determined based on the maximal Youden’s index on the ROC analysis of the non-paired SSTR PET/CT SUV_max_ values in the hilar and mediastinal nodes in patients with TC. To compare SUV_max_ values in metastatic lesions on SSTR PET/CT and on [^18^F]FDG PET/CT, a linear mixed model was used for data analysis, including a random intercept to account for clustering by paired data. The analysis was performed on a log-transformed outcome variable. All tests were performed as two-sided tests, and p values of less than 0.05 were considered significant. The analyses were carried out through IBM SPSS Statistics (version 27) and SAS software (version 9.4 of the SAS System for Windows).

### Literature search

A literature search was performed using The National Center for Biotechnology Information PubMed online database. The following key words were used for selection of studies: “pulmonary carcinoid” or “bronchial carcinoid” AND “DOTA PET”. The references from retrieved papers were also searched for suitable publications. Ten publications were deemed suitable based on the following inclusion criteria: original research, SSTR PET/CT performed for pathologically proven PC and number of patients five or more.

## Results

A total of 87 patients were withheld from the databases. One patient was excluded because of the diagnosis of a primary pulmonary paraganglioma on final pathological evaluation, yielding a total of 86 patients with pulmonary NETs. Patient and tumour characteristics are listed in Table 1. Nearly two-thirds of the patients were female (55/86; 64%), and the median age of the patient population was 60 years (range 15–84).

**Table 1:** Patient and tumour characteristics

Variable	Count (N)/Value	Percentage (%)
Sex		
Male Female	31 55	36.0 64.0
Age (y)		
Mean Median	54.x 60.x	- -
Tracer		
[^68^Ga]Ga-DOTATOC [^68^Ga]Ga-DOTATATE	26 60	30.2 69.8
[^18^F]FDG PET/CT		
Yes No	46 40	53.5 46.5
Tumour type (resection/biopsy)		
TC AC LCNEC Carcinoid NOS	62 19 3 2	72.1 22.1 3.5 2.3
Tumour location		
Trachea/main bronchi Right upper lobe Right middle lobe Right lower lobe Left upper lobe Left lower lobe Other	7 11 12 25 11 11 9	8.2 12.8 14.0 29.1 12.8 12.8 10.5
Resection primary tumour		
Yes, after scan Yes, before the scan No	51 16 19	59.3 18.6 22.1
N stage		
pN0 pN1 pN2 pN3 Nx	47 4 9 3 23	54.7 4.7 10.5 3.5 26.7

Sixty-two patients (72%) underwent the [^68^Ga]Ga-SSTR PET/CT during primary staging of the PC, 2 patients (2%) during screening for NETs in the context of adrenocorticotropic hormone (ACTH) overproduction, 3 patients (4%) in the context of increased serum chromogranin levels after resection of a PC in the past, and 19 patients (22%) during follow-up for metastatic disease. The most frequent location of the primary tumour was the lower lobe of the right lung. Forty-nine of the 62 patients in the staging group (79%) and each of the 2 patients in the ACTH screening group underwent surgical resection of the primary tumour, yielding the diagnosis of TC in 43 patients, of AC in 7 patients and of LCNEC in 1 patient. Of the patients that underwent the SSTR PET/CT after resection of a PC in the past (*N* = 16), 8 had a primary TC, 6 a primary AC and 2 a carcinoid not otherwise specified (NOS). Taken together, resection specimens revealed 51 TCs, 13 ACs, 1 LCNEC and 2 carcinoids NOS in total. In 19 patients, no resection of the primary tumour occurred but pathology of the tumour and/or metastatic lesions was available through biopsy and was suggestive for TC in 11 patients, for AC in 6 patients and for LCNEC in 2 other patients.

**Fig. 1:**
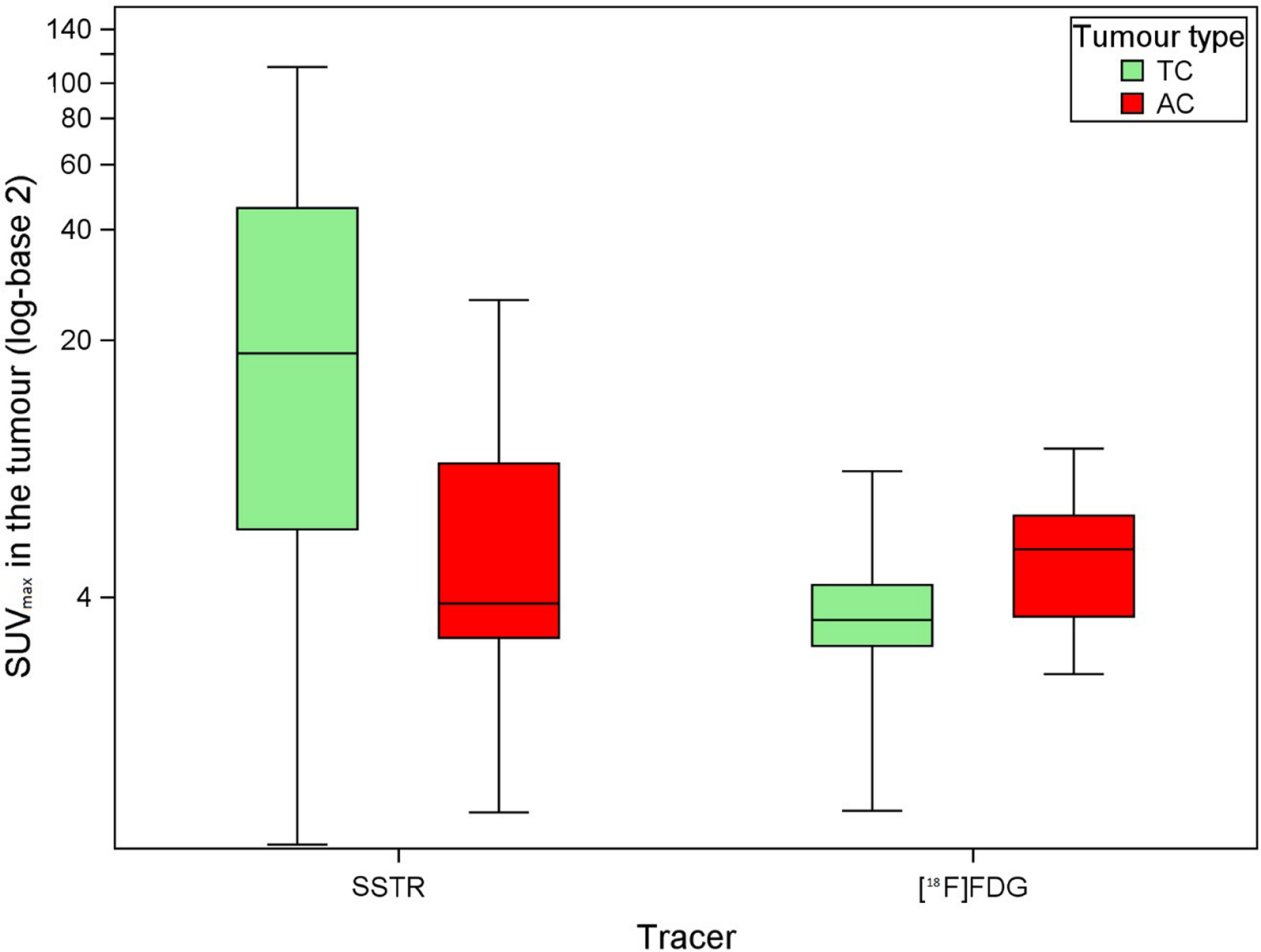
Box plots of the distribution of SUV_max_ values in function of tracer and tumour type, yielding a median SUV_max_ on SSTR PET/CT of 18.4 and 3.8 for 52 typical bronchial carcinoid tumours and 12 atypical bronchial carcinoid tumours, respectively, and a median SUV_max_ on [^18^F]FDG PET/CT of 3.5 and 5.4 for 28 typical bronchial carcinoid tumours and 9 atypical bronchial carcinoid tumours, respectively

**Fig. 2:**
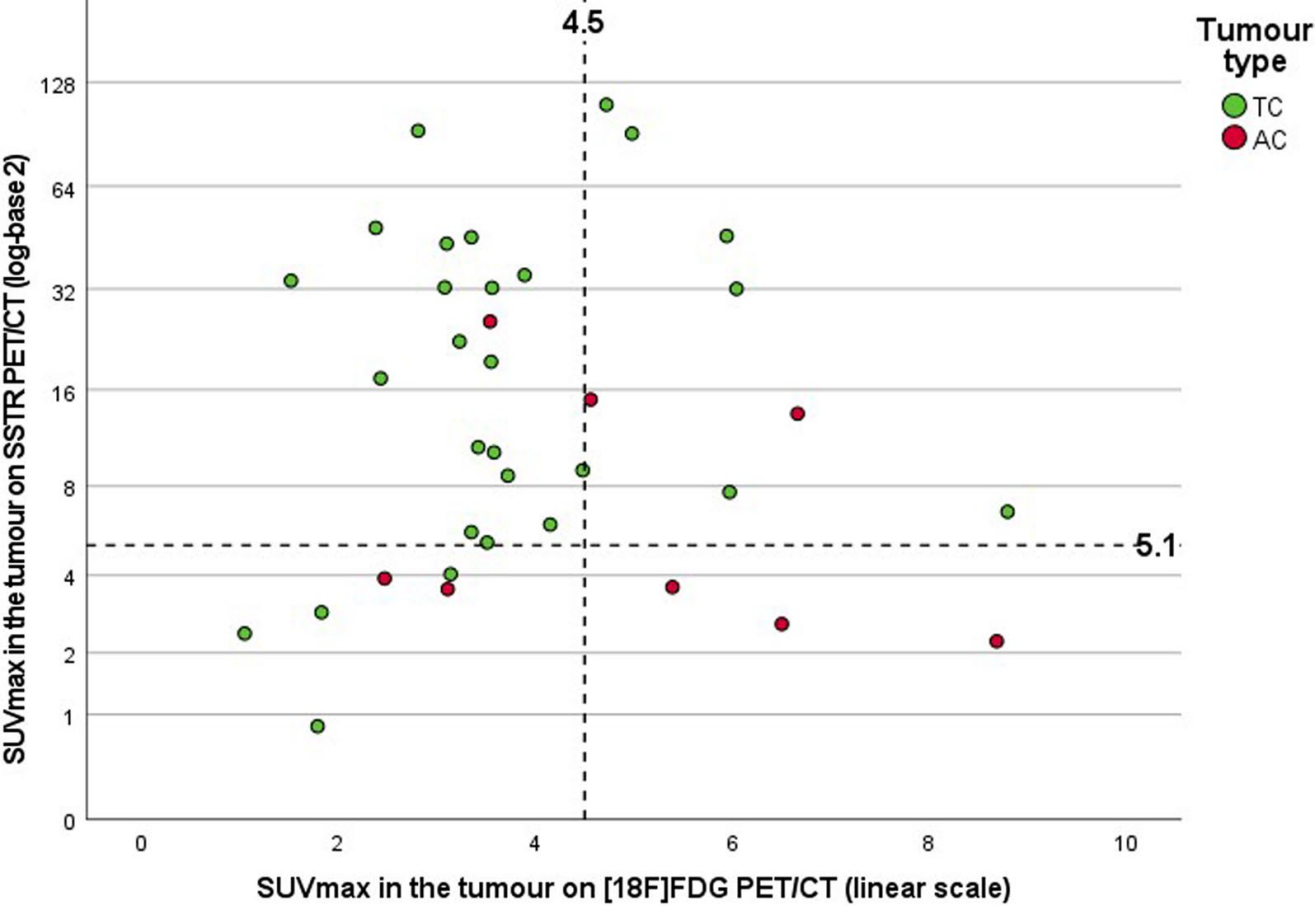
Scatter plot of the paired SUV_max_ values on SSTR PET/CT and [^18^F]FDG PET/CT scans for typical (TC) and atypical (AC) bronchial carcinoid tumours (n = 36), with dashed reference lines on the cut-off SUV_max_ values (5.1 for SSTR PET/CT and 4.5 for [^18^F]FDG PET/CT) based on the maximal Youden’s index derived from the ROC curves of the non-paired SUV_max_ values on SSTR and [^18^F]FDG PET/CT scans for TC and AC, respectively (see Additional file figures)

The primary tumour was in situ when performing the SSTR PET/CT in 70 of the 86 patients. However, 6 more patients were excluded for the SUV_max_ analysis in the primary tumour because of the absence of pathologic sampling of the tumour (*n* = 4), because of the inability to delineate the primary tumour (*n* = 1) or because of the pathological diagnosis of a LCNEC in a tumour with low tracer uptake (*n* = 1), yielding 64 primary tumours (52 TC and 12 AC tumours) available for pathology-based SUV_max_ analysis (see the Consolidated Standards of Reporting Trials (CONSORT) diagram in Additional file 1: Fig. S1). The median SUV_max_ value of all pathologically proven TC tumours was 18.4 (range 0.9–110.2) on SSTR PET/CT and 3.5 (range 1.1–8.8) on [^18^F]FDG PET/CT (Fig. 1). For AC tumours, the median SUV_max_ value was 3.8 (range 1.0–25.7) on SSTR PET/CT and 5.4 (range 2.5–10.1) on [^18^F]FDG PET/CT. The difference in tumoural SUV_max_ between TC and AC was statistically significant for SSTR PET/CT (*p* = 0.003) as well as for [^18^F]FDG PET/CT (*p* = 0.038). ROC curves were generated for non-paired values of tumoural SUV_max_, resulting in an AUC of 0.78 for the detection of TC on SSTR PET/CT and an AUC of 0.73 for the detection of AC on [^18^F]FDG PET/CT (Additional file 1: Fig. S2). These ROC curves were used to calculate the maximal Youden’s index, occurring at a cut-off point of SUV_max_ 5.1 for TC on SSTR PET/CT and at a cut-off point of SUV_max_ 4.5 for AC on [^18^F]FDG PET/CT. Division of the scatter plot distribution of paired tumoural SUV_max_ values in quadrants based on these cut-off values shows that the majority of TC tumours are seen in the upper left quadrant, which represents low [^18^F]FDG and high SSTR ligand uptake, with 18 out of 19 tumours (95%) in this quadrant being TC. No TC tumours were found in the lower right quadrant, which represents high [^18^F]FDG and low SSTR ligand uptake, with all 3 out of 3 tumours (100%) in this quadrant being AC (Fig. 2). The probability to find an AC in the right upper quadrant and the left lower quadrant was 2/8 (25%) and 2/6 (33.3%), respectively, in line with the pretest probability for a tumour being AC (19/86; 22.1%, Table 1).

**Fig. 3:**
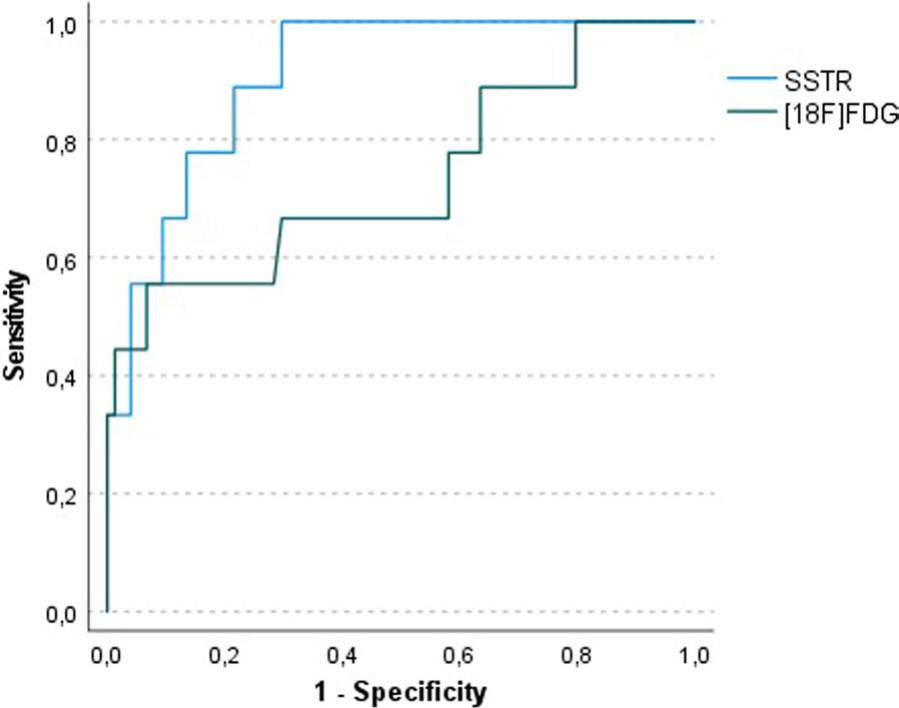
ROC curve analysis of paired SUV_max_ values of hilar/mediastinal lymph node stations of typical bronchial carcinoid tumours (TC) on SSTR PET/CT (blue curve) and [^18^F]FDG PET/CT (green curve), yielding an area under the curve (AUC) of 0.91 and 0.74, respectively. The difference between the AUC values for SSTR and [^18^F]FDG was 0.17 (*p* = 0.10)

Of all the patients that underwent a resection of the primary tumour with concurrent hilar and mediastinal lymph node dissection (*N* = 63)—both after and prior to the SSTR PET/CT—the diagnosis of N0-disease was made in 47 patients (75%), of N1-disease in 4 patients (6.3%), of N2-disease in 9 patients (14%) and of N3-disease in 3 patients (4.8%). Pathology of hilar and/or mediastinal lymph node stations was available and could be correlated with SUV_max_ values on SSTR PET/CT in all 51 patients that underwent a resection of the tumour after the SSTR PET/CT. [^18^F]FDG PET/CT and thus paired SSTR ligand/[^18^F]FDG SUV_max_ values in the nodes were available in 29 of these 51 patients (57%). In 4 of the patients of whom the primary tumour detected on SSTR PET was not resected, hilar and/or mediastinal lymph node stations were pathologically examined through mediastinoscopy (*N* = 3) or endobronchial ultrasound-guided transbronchial needle aspiration (EBUS-TBNA, *N* = 1). In 2 of these 4 patients, paired SSTR ligand/[^18^F]FDG SUV_max_ values could be obtained (CONSORT in Additional file 1: Fig. S3). In one other patient, although the SSTR PET/CT scan was performed after resection of the primary tumour, an [^18^F]FDG PET/CT was carried out prior to this resection, with recording of [^18^F]FDG SUV_max_ values in the lymph node stations that were pathologically evaluated through this resection. Taken together, a comparison of 267 hilar and/or mediastinal lymph node stations in these 56 patients was retrospectively conducted between pathological analysis (TC/AC; *N* +/N−) and preoperative imaging data (SSTR ± [^18^F]FDG PET/CT). Paired SUV_max_ values on both [^18^F]FDG and SSTR PET were noted in 103 (83 TC and 20 AC) of these 267 lymph node stations, resulting in an AUC for the detection of metastatic hilar/mediastinal lymph nodes in TC of 0.91 for SSTR PET/CT and of 0.74 for [^18^F]FDG PET/CT, with a difference of 0.17 (95% confidence interval (CI) -0.03 to 0.38; *p* = 0.10) (Fig. 3). ROC curves of non-paired SUV_max_ values in the 267 hilar and mediastinal lymph node stations of TC and AC were generated for both SSTR PET/CT and [^18^F]FDG PET/CT (Additional file 1: Fig. S4), showing an AUC of 0.82 for the detection of lymph node metastasis of TC on SSTR PET/CT and an AUC of 0.93 for the detection of lymph node metastasis of AC on [^18^F]FDG PET/CT. Based on the ROC analysis of the non-paired SUV_max_ values in the 167 nodes of TC on SSTR PET/CT, we derived a maximal Youden’s index at SUV_max_ 2.1 using resection specimens as well as biopsies for pathological evaluation, resulting in a sensitivity and specificity for regional lymph node involvement of TC on SSTR PET/CT of 80% and 75%, respectively (Table 2). Interestingly, all pathologically examined regional lymph node stations with an SUV_max_ of 4.0 or more on SSTR PET/CT turned out to be metastatic, yielding a true positive rate of 100% at this SUV_max_ cut-off value.

**Fig. 4:**
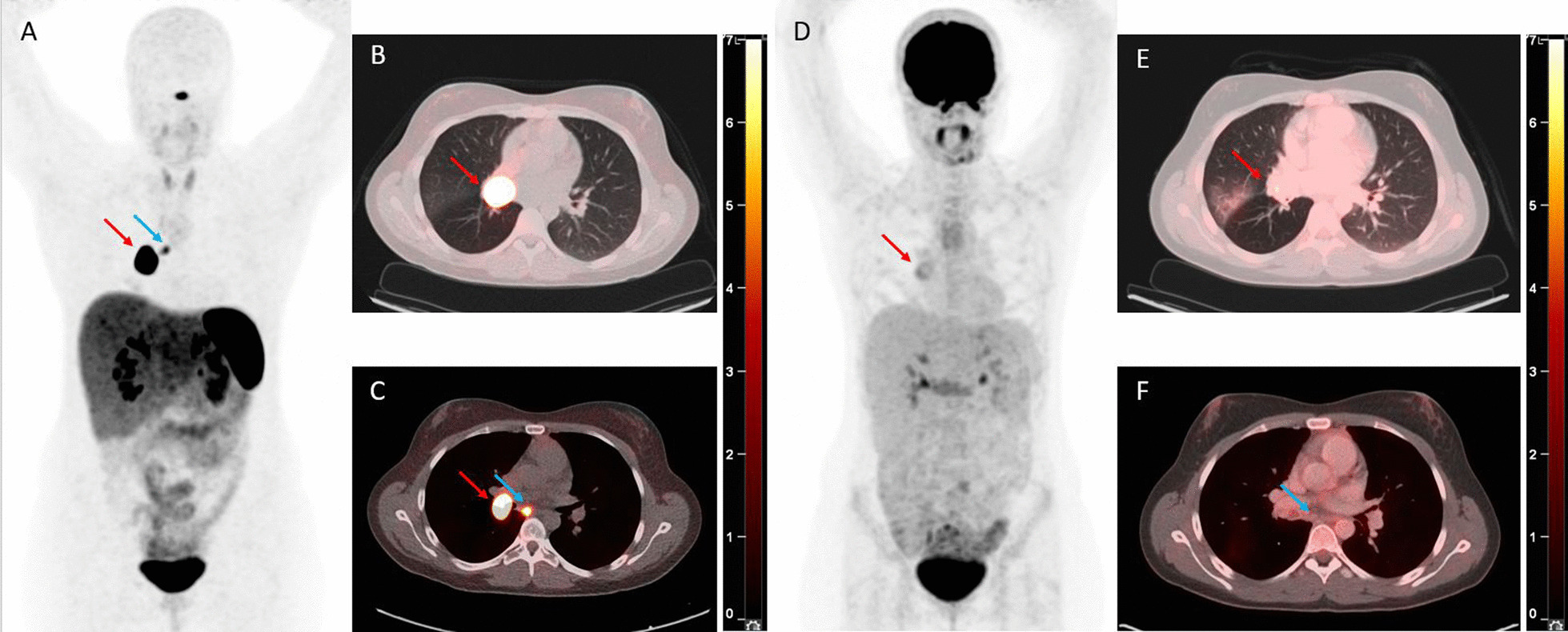
21-year-old patient with a typical bronchial carcinoid (TC) who underwent a [^68^Ga]Ga-DOTATOC PET/CT scan showing two foci of intense tracer uptake on the MIP image (A) corresponding to the primary hilar tumour (red arrow, B) and to an infracarinal nodal metastasis (blue arrow, C), as well as an [^18^F]FDG PET/CT scan showing only a limited tracer uptake in the tumour (red arrow, visible on the MIP image (A) and an axial fusion image (E)) and no increased tracer uptake in the infracarinal nodal metastasis (blue arrow, F). The SUV_max_ values on [^68^Ga]Ga-DOTATOC PET/CT were 110 and 9.2 in the tumour and in the infracarinal lymph node, respectively, whereas those on [^18^F]FDG PET/CT were 4.7 and 2.3 in the tumour and the infracarinal lymph node, respectively

**Table 2:** 2 × 2 contingency table for the evaluation of nodal disease of TC on SSTR PET/CT using an SUV_max_ cut-off value of 2.1

Nodal disease (TC)	Histopathology +	Histopathology −
SSTR PET/CT +	16	37
SSTR PET/CT −	4	110

Twelve lesions suspicious for distant metastatic disease seen on SSTR PET/CT in 10 patients were biopsied (CONSORT in Additional file 1: Fig. S5) with pathological confirmation of metastatic disease in all of these lesions, yielding a positive predictive value (PPV) of 100% in this small sub-cohort of patients with pathological validation (Table 3). The median SUV_max_ value of metastases was 12.7 on SSTR PET and 2.6 on [^18^F]FDG PET. Analysis based on a linear mixed model for paired as well as non-paired SUV_max_ values showed significantly higher SUV_max_ values on SSTR PET in comparison with [^18^F]FDG PET (*p* = 0.006), regardless of the tumoural entity (TC versus AC).

**Fig. 5:**
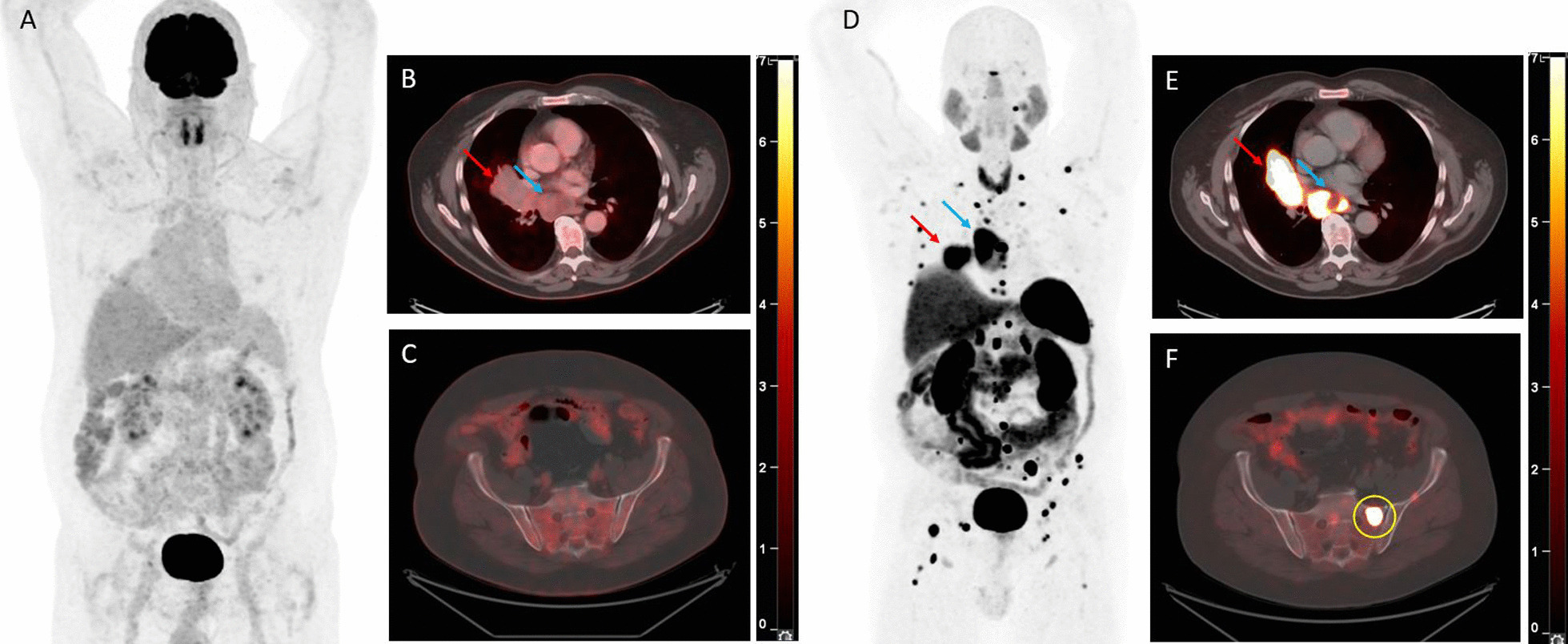
MIP image of an [^18^F]FDG PET/CT scan (A) and a [^68^Ga]Ga-DOTATATE PET/CT scan (D) performed during staging of a typical bronchial carcinoid tumour (TC) of a 62-year-old patient with a clear discrepancy between an intense [^68^Ga]Ga-DOTATATE uptake (E) and no increased [^18^F]FDG uptake (B) in the primary tumour (red arrow) as well as in a subcarinal lymph node metastasis (blue arrow). The axial fusion images with CT in bone window of the [^18^F]FDG PET/CT (C) and the [^68^Ga]Ga-DOTATATE PET/CT (F) also show this discrepancy with regard to the bone metastases. The bone metastasis in the left hemisacrum (yellow circle) has a SUV_max_ on [^68^Ga]Ga-DOTATATE PET/CT of 39.6. A chest CT 53 days before the [^68^Ga]Ga-DOTATATE PET/CT did not show any bone metastasis

**Table 3:** All lesions suspicious for metastases on SSTR PET/CT that were pathologically examined were confirmed as metastases of bronchial NETs

Patient case	Pathology	Localization metastasis		SUV_max_ SSTR PET/CT	SUV_max_ [^18^F]FDG PET/CT
1	TC	Supraclavicular node	3.49		–
2	TC	Breast	4.50		1.80
3	TC	Sternum	5.63		–
4	TC	Subcutaneous nodule	7.28		–
2	TC	Paravertebral node	9.97		2.50
5	TC	Liver	29.6		–
6	TC	Liver	31.5		–
7	TC	Liver	36.6		5.68
2	TC	Liver	49.3		3.10
8	Carcinoid NOS	Parotid gland	34.8		2.13
9	AC	Rib 10	4.55		–
10	AC	Liver	10.3		4.44

## Discussion

In this second largest series since the introduction of SSTR PET/CT (Table 4), we retrospectively evaluated clinical, pathological and imaging data of 86 patients diagnosed with pulmonary NETs. There was a predominance of TC over AC (and LCNEC) and of female over male patients, consistent with epidemiologic data in the literature concerning these tumour types [5, 37, 38]. Interestingly, in 2 of the 51 patients that underwent surgical resection after the SSTR PET/CT, the diagnosis of PC was made during screening in the context of increased ACTH secretion, consistent with the recent literature evaluating the role of SSTR PET/CT in the detection of ectopic ACTH-secreting tumours [39, 40].

**Table 4:** Literature overview of [^68^Ga]Ga-peptide PET/CT in pulmonary carcinoid tumours (PCs) (case reports excluded)

Author	Year	n	[^68^Ga]Ga-peptide	Main results
Kumar [41]	2009	7	-DOTATOC	TCs had mild [^18^F]FDG uptake and high [^68^Ga]Ga-DOTATOC uptake. ACs had moderate uptake of [^18^F]FDG and high [^68^Ga]Ga-DOTATOC uptake. The combined use of [^18^F]FDG and [^68^Ga]Ga-DOTATOC PET/CT reveals different uptake patterns in various bronchial tumours
Ambrosini [24]	2009	11	-DOTANOC	[^68^Ga]Ga-DOTANOC PET/CT provided additional information in 9 of 11 patients compared to conventional imaging, leading to changes in the clinical management of 3 of these 9 patients
Kayani [39]	2009	18	-DOTATATE	Typical bronchial carcinoids showed higher and more selective uptake of [^68^Ga]Ga-DOTATATE than of [^18^F]FDG. Atypical carcinoids and higher grades had less [^68^Ga]Ga-DOTATATE avidity but were [^18^F]FDG-avid
Jindal [43]	2011	20	-DOTATOC	TCs had a lower [^18^F]FDG and a higher [^68^Ga]Ga-DOTATOC uptake compared with ACs. The ratio of SUVmax on [^68^Ga]Ga-DOTATOC and on [^18^F]FDG PET/CT was a better predictor of the histopathologic variety of the PC compared with the SUV_max_ on the 2 types of scans individually
Venkitaraman [44]	2014	32	-DOTATOC	[^68^Ga]Ga-DOTATOC has a high sensitivity, specificity and accuracy in the detection of PC, whereas [^18^F]FDG PET/CT suffers from a low sensitivity and specificity in differentiating PCs from other tumours
Lococo [10]	2015	33	-DOTATOC -DOTATATE -DOTANOC	[^68^Ga]Ga-DOTA-peptide PET/CT was superior in detecting TC whereas [^18^F]FDG PET/CT was superior in detecting AC. The SUVmax ratio was the most accurate semiquantitative index in identifying TC
Prasad [49]	2015	27	-DOTATOC -DOTATATE	It is necessary to combine functional ([^68^Ga]Ga-SSR PET) and morphological imaging in the restaging of patients with TC and AC. The major advantage of [^68^Ga]Ga-SSR PET lies in the detection of additional bone lesions
Lococo [45]	2019	26	-DOTATOC	In the detection of PCs, [^68^Ga]Ga-DOTATOC PET ensures better diagnostic performance compared to [^18^F]FDG PET. [^68^Ga]Ga-DOTATOC performs at its best in TCs, and [^18^F]FDG in ACs. [^68^Ga]Ga-DOTATOC uptake was negatively correlated with the number of mitoses and the presence of necrosis
Komek [46]	2019	20	-DOTATATE	SUV_max_ values were higher for atypical PC on [^18^F]FDG PET and for typical PC on [^68^Ga]Ga-DOTATATE PET, indicating the potential utility of the SUV_max_ ratio in predicting the histological subtype of PC tumours
Purandare [11]	2020	119	-DOTANOC	[^68^Ga]Ga-DOTANOC PET/CT is highly sensitive in detecting PC and detects asymptomatic distant metastatic disease in a sizeable number of patients (11.7%), thus contributing to clinical management. TCs show significantly higher uptake than ACs. [^68^Ga]Ga-DOTA-peptide PET/CT should be an integral part of the diagnostic work-up of patients with PC
Deleu (this series)	2022	86	-DOTATOC -DOTATATE	The role of PET/CT in the assessment of the tumour biology of PC was confirmed based on a significantly higher SSTR ligand and lower [^18^F]FDG uptake in TC compared to AC. Moreover, a high sensitivity of 80% of SSTR PET/CT in detecting regional lymph node metastases was found. Finally, SSTR PET/CT has a PPV of 100% in a small sub-cohort of patients with pathologically examined distant metastases

While the European guidelines from the European Society for Medical Oncology (ESMO) and the European Neuroendocrine Tumor Society (ENETS) recommend performing SSTR PET/CT in addition to contrast-enhanced CT in the TNM staging of PC, recent Commonwealth and North American guidelines also endorse SSTR PET/CT in the detection of metastatic disease, but suggest only a limited clinical utility of SSTR PET in detecting metastases in patients with a small primary PC [1, 2, 4]. These guidelines are based on a few small to medium-sized studies that evaluated the role of SSTR PET/CT in the staging of PC, as seen in our literature review in Table 4. In 2009, Kumar et al., Ambrosini et al. and Kayani et al. were the first to describe the additional value of SSTR PET/CT in lung NET, changing the clinical management of patients with PC [23, 41–43]. Together with additional series from 2011 until 2020, all authors observed a high SSTR and low [^18^F]FDG uptake in TC and vice versa for AC, known as the “flip-flop phenomenon” due to the presence of specific molecular markers in the more benign tumours and loss of neuroendocrine markers with increased glycolytic phenotype in the more aggressive tumours [31]. This highlights the value of dual tracer PET/CT in the preoperative assessment of tumour biology [10, 44–47]. Our study confirms these findings by demonstrating a significant difference in tumoural SUV_max_ values between TC and AC on SSTR as well as on [^18^F]FDG PET/CT, underscoring the role of SSTR PET in low grade PC and of [^18^F]FDG PET/CT in intermediate grade PC (Fig. 1). For this pathology-based analysis, we relied mostly (54/64; 84%) on resection specimens, in accordance with recent guidelines for diagnosis and management of patients with lung neuroendocrine tumours [2]. Our median SUV_max_ values in the tumoural lesion are in line with the values reported in the studies listed in Table 4. Based on the literature and our current findings, we suggest to use SSTR PET as first-line molecular imaging test for biopsy-proven TC, whereas for AC the choice between SSTR PET and [^18^F]FDG PET could be made based on tumour biology (e.g. if high Ki-67 index or mitotic count, start with [^18^F]FDG). In case of low tracer avidity, additional imaging with the other tracer can be considered.

Furthermore, this is—to the best of our knowledge—the first study in which SUV_max_ values in hilar and mediastinal lymph node stations on SSTR PET/CT were correlated with nodal involvement on pathologic evaluation. We found an AUC of 0.91 on the ROC curve for the detection of regional lymph node metastases from TC on SSTR PET/CT. Based on the maximal Youden’s index of this ROC analysis, an associated sensitivity and specificity of 80% and 75%, respectively, was found. However, a rather high rate of false-positive findings at the SUV_max_ cut-off of 2.1 should be kept in mind when interpreting lymph nodes on SSTR PET/CT, entailing the need to confirm lymph node metastases on SSTR PET/CT that would render the patient inoperable by biopsy. If an SUV_max_ cut-off value of 4.0 was applied, the true positive rate was 100%. Given the crucial role of complete resection of lymph node metastases as well as the tendency for surgeons to perform minimally invasive (sub)lobar tumour resections with more limited lymph node assessment, SSTR PET/CT could be seen as a noninvasive way to determine N status and to guide lymph node dissection in these tumour types [4, 32, 38, 48, 49]. In our series as well, 14/51 patients (27%) who underwent a surgical lymphadenectomy after a staging or screening (in the context of ACTH overproduction) SSTR PET/CT presented with pathologically proven N + stage (cfr example in Fig. 4). [^18^F]FDG PET/CT also provides a good diagnostic performance in the detection of regional lymph node metastasis in AC (AUC 0.93); however, these data should be interpreted with caution given the small cohort of 11 lymph nodes evaluated for this more uncommon tumour type. The European Society for Thoracic Surgery (ESTS) performed an electronic survey on the surgical management of PCs in 2012, with responses from 172 institutions worldwide. The responders agreed that, if the primary tumour is resectable and if nuclear imaging suggests N2M0 disease, surgery can be performed with no need for further preoperative invasive staging [1].

Finally, regarding detection of metastatic disease, the additional value of SSTR PET/CT is observed in many publications, especially for the detection of bone metastases. In a large series of postoperative surveillance in 337 patients with resected PC, routine CT scan of the chest and upper abdomen—the recommended imaging modalities during follow-up—failed to detect recurrences in 15 of the 20 patients (75%) with distant recurrence [25]. However, the literature on the quantitative evaluation of the added value of SSTR PET/CT is scarce with only two studies assessing this hypothesis. Prasad et al. and Purandare et al. were able to prove the contribution of SSTR PET/CT to the clinical management of patients with metastatic lesions of PC by precluding futile surgeries in 10 to 15% of patients, and by accurately detecting metastases during restaging (Table 4) [11, 50]. In our study, pathological examination of lesions suspicious for metastases during staging or follow-up SSTR PET/CT was obtained in 12 lesions of 10 patients and was positive for metastasis of PC in all lesions, yielding a positive predictive value (PPV) of 100% for SSTR PET/CT in this small sub-cohort of patients with pathologically evaluated lesions (Table 3). An illustration of a patient from our series with M0 disease on staging CT, but with the diagnosis of multifocal bone metastases on [^68^Ga]Ga-DOTATATE PET/CT, is shown in Fig. 5, changing the therapeutic approach from curative-intent surgery to non-curative systemic treatment. Although the bone metastases were not pathologically proven in this patient, the SUV_max_ of 39.6 in the most intense lesion was highly suggestive, and follow-up imaging confirmed the M1 stage.

One of the major limitations of this analysis is the retrospective nature and the potential selection bias due to the fact that patients who were referred for an SSTR PET/CT could have a higher probability for relapse during restaging or for metastatic disease during staging. Also the fact that the scans were performed at a single university reference centre may contribute to this selection bias. Furthermore, we used two different [^68^Ga]Ga-peptides and three different PET/CT cameras. Although the tracers have slightly different binding affinities to SSTR subtypes and the different PET/CT cameras improved in performance over the years, there seems to be no clinically relevant difference in the diagnostic accuracy. We included SSTR scans performed in the context of tumour screening, staging, restaging, and therapy evaluation, representing a heterogenous clinical setting. Given the inclusion of 19 patients during follow-up, a possible influence of previous therapies on the SUV_max_ values cannot be excluded. However, this heterogeneity also provides a cross-section of the indications to perform a SSTR PET/CT and resulted in a total cohort of 86 patients, representing the second largest series of SSTR PET/CT in patients with PC in the literature. This was necessary to obtain a sufficiently large number of lesions for the pathological correlation of regional lymph node involvement as well as for the pathological validation of hematogenous metastases. We acknowledge the possibility of discordance between the nodes examined by the pathologist and the localization of these nodes on the preoperative SSTR PET/CT. Finally, in the minority of cases where no resection specimen was available (10/64 (16%) SSTR PET/CTs for tumoural SUV_max_ analysis and 4/56 (7%) SSTR PET/CTs for nodal SUV_max_ analysis), pathological data from biopsies were also included, which could have led to a slight overdiagnosis of TC that would have been classified as AC after resection.

## Conclusion

In this study, the performance of SSTR PET/CT in patients with pulmonary neuroendocrine (carcinoid) tumours was studied in a large series with pathology as gold standard. Our results confirm the important role of PET/CT in the assessment of the tumour biology, based on a significant higher SSTR ligand uptake and lower [^18^F]FDG uptake in TC compared to AC. Moreover, the assessment of the diagnostic performance of SUV_max_ values in pathologically evaluated hilar and mediastinal lymph node stations revealed a high diagnostic accuracy of SSTR PET/CT for regional lymph node metastases of TC. Finally, SSTR PET/CT has a PPV of 100% in patients with pathologically examined metastatic lesions, albeit in a small sub-cohort. Therefore, our data lend support to the current European guidelines (ESMO and ENETs) that recommend first-line conduct of SSTR PET/CT in the staging and restaging of pulmonary NETs.

## Supplementary Information

**Additional file 1.** Supplementary Figures.

## Data Availability

The data sets generated and/or analysed during the current study are not publicly available due patient confidentiality reasons but are available from the corresponding author on reasonable request and pending approval from the Ethics Committee of the University Hospitals of Leuven.
