# Supplementary material for: Value of [68Ga]Ga-somatostatin receptor PET/CT in the grading of pulmonary neuroendocrine (carcinoid) tumours and the detection of disseminated disease: single-centre pathology-based analysis and review of the literature
Source: EJNMMI Res. 2022 May 7;12:28. doi: 10.1186/s13550-022-00900-3 (PMC9079198; doi:10.1186/s13550-022-00900-3)
Supplement: Supplementary file 1 — Additional file 1. Supplementary Figures. [file 13550_2022_900_MOESM1_ESM.docx]

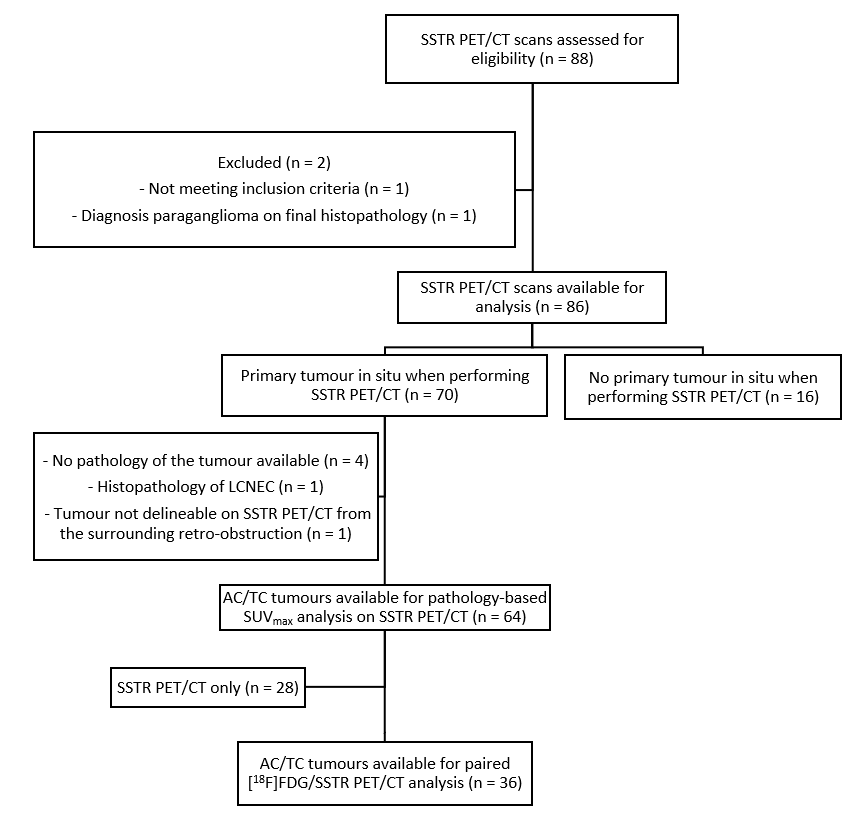


**Supplemental figure 1** Consolidated Standards of Reporting Trials (CONSORT) diagram for tumoural SUV_max_ analysis


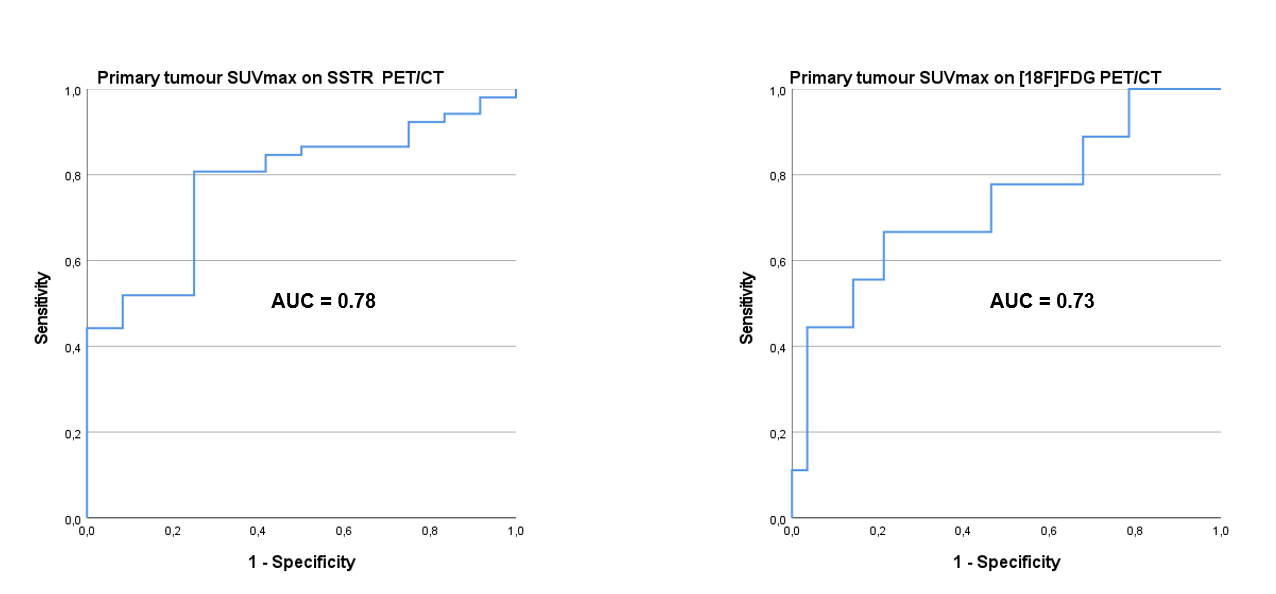


**Supplemental figure 2** ROC curve analysis of the sensitivity of non-paired SUV_max_ values in detecting TC on SSTR PET/CT (left curve) and AC on [^18^F]FDG PET/CT (right curve), yielding a maximal Youden’s index for detecting TC on SSTR PET/CT at SUV_max_ of 5.1 and for detecting AC on [^18^F]FDG PET/CT at SUV_max_ of 4.5 (see dashed reference lines on figure 2)


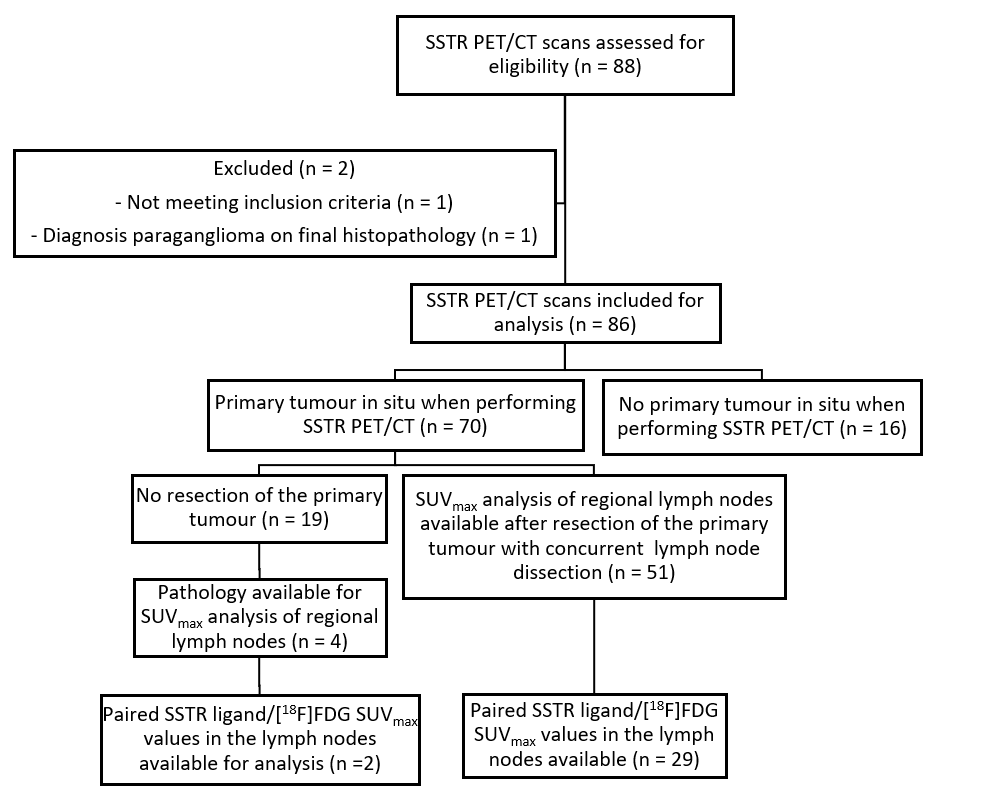


**Supplemental figure 3** Consolidated Standards of Reporting Trials (CONSORT) diagram for SUV_max_ analysis of regional lymph nodes


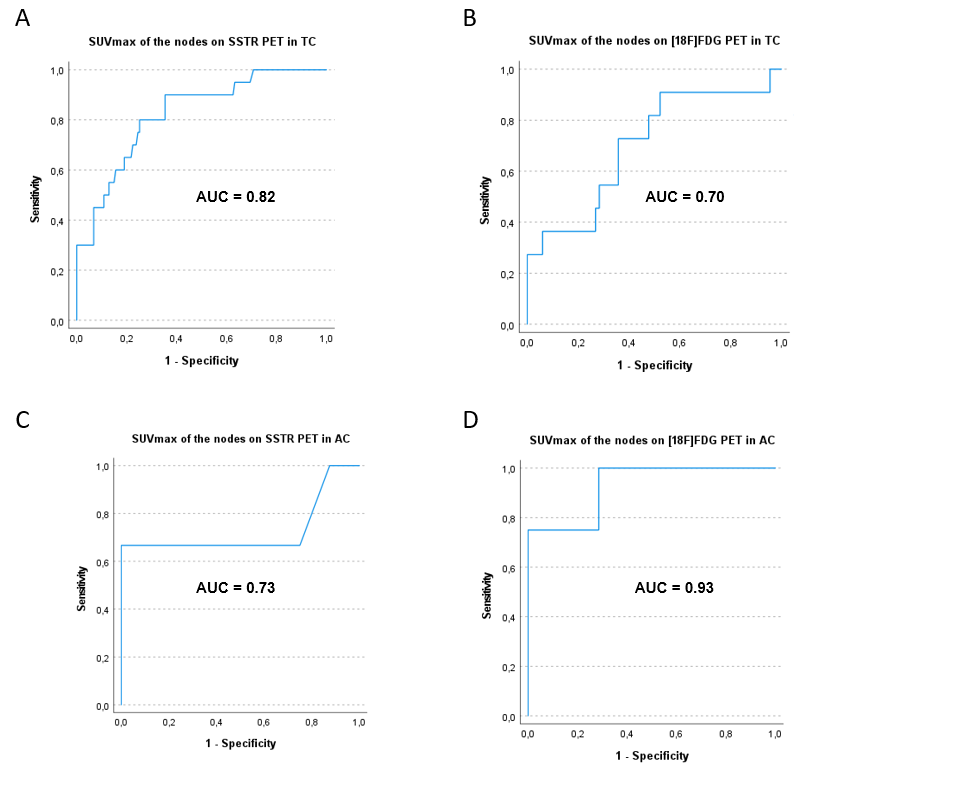


**Supplemental figure 4** ROC curve analyses of non-paired SUV_max_ values of mediastinal lymph node stations of typical bronchial carcinoid tumours (TC, upper 2 curves) and atypical bronchial carcinoid tumours (AC, lower 2 curves) with pathological examination as gold standard, reflecting the higher diagnostic accuracy for the detection of mediastinal lymph node metastases of SSTR PET/CT in comparison to [^18^F]FDG PET/CT for TC. A: ROC curve analysis of SUV_max_ values on SSTR PET/CT of 167 mediastinal lymph node stations of TC, yielding an area under the curve (AUC) of 0.82. B: ROC curve analysis of SUV_max_ values on [^18^F]FDG PET/CT of 78 mediastinal lymph node stations of TC, yielding an AUC of 0.70. C: ROC curve analysis of SUV_max_ values on SSTR PET/CT of 11 mediastinal lymph node stations of AC, yielding an AUC of 0.73. D: ROC curve analysis of SUV_max_ values on [^18^F]FDG PET/CT of 11 mediastinal lymph node stations of AC, yielding an AUC of 0.93.


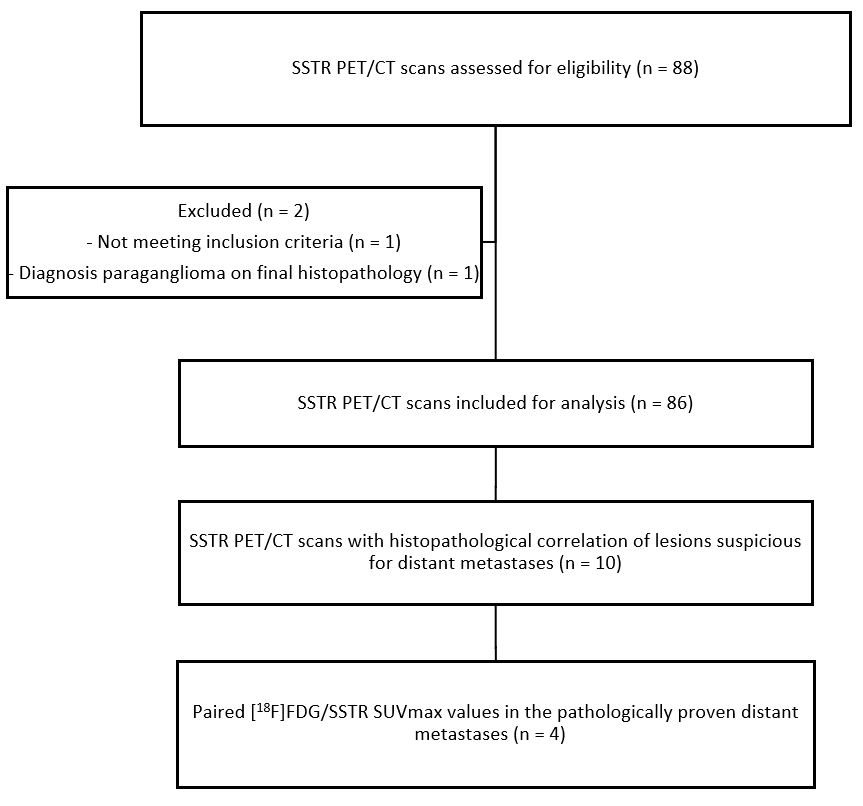


**Supplemental figure 5** Consolidated Standards of Reporting Trials (CONSORT) diagram for SUV_max_ analysis of pathologically proven distant metastatic lesions.

|  |
| --- |
